# The Association of Internet Use with Wellbeing, Mental Health and Health Behaviours of Persons with Disabilities

**DOI:** 10.3390/ijerph16183252

**Published:** 2019-09-04

**Authors:** Mariusz Duplaga, Katarzyna Szulc

**Affiliations:** 1 Department of Health Promotion, Faculty of Health Sciences, Institute of Public Health, Jagiellonian University Medical College, 31-008 Kraków, Poland; 2 Scientific Student’s Circle of Health Promotion, Department of Health Promotion, Institute of Public Health, Faculty of Health Sciences, Jagiellonian University Medical College, 31-008 Kraków, Poland

**Keywords:** disability, digital divide, Internet use, wellbeing, mental health, health behaviours

## Abstract

Introduction: There is strong evidence that people with disabilities suffer from a significant digital divide. However, there are reports indicating that Internet use may result in many benefits for those with disabilities. The aim of the study was to assess the impact that the use of the Internet has on the wellbeing and health behaviours of persons with disabilities. Methods: An analysis was carried out using the dataset obtained from Social Diagnosis, a panel study undertaken on a nationally representative sample. The records of persons with disabilities were retrieved from the dataset which was established in 2015. An analysis of the association between Internet use and the wellbeing, mental health and health behaviours of the respondents was undertaken. The variables reflecting the self-assessment of their own life and experience of loneliness were treated as being indicators of their wellbeing and the prevalence of suicidal thoughts or making use of psychological help as indicators of mental health. The health behaviours analysed in the study included smoking, excessive consumption of alcohol and undertaking physical activity. For all these variables, multivariate logistic regression models were developed. The effect of Internet use was adjusted for sociodemographic variables and the degree of disability. An analysis was performed after applying post-stratification weights available from the Social Diagnosis study. Results: The weighted study group consisted of 2529 people having a mean age of 59.33 ± 16.89 years. The group included 20.71% (N = 524) respondents with a mild, 41.58% (N = 1052) with a moderate, and 26.54% (N = 671) with a severe disability. The proportion of Internet users was 37.07% (N = 937). In all the regression models, Internet use had a significant impact on the dependent variables. After adjustment for sociodemographic variables and the degree of disability, the Internet users more frequently assessed their lives as happy (odds ratio, 95% confidence interval: 1.86, 1.47–2.37) and less frequently experienced loneliness (0.63, 0.49–0.81) or suicidal thoughts (0.47, 0.35–0.65). In addition, they needed psychological help less frequently (0.50, 0.35–0.72). Interestingly, Internet users undertook some form of physical activity or sport more often (2.41, 1.87–3.13) and fewer smoked cigarettes (0.70, 0.54–0.91) or consumed alcohol excessively (0.32, 0.19–0.56). Conclusions: The use of the Internet by people with disabilities was associated with improved wellbeing, better mental health and more beneficial health behaviours. These findings support the development of intensive actions to reduce the digital divide for the population of people with disabilities.

## 1. Introduction

Disability is an umbrella term used to include impairments, limitations to an individual’s activity and restriction to participation [1]. The understanding of the term has changed radically since the 1970s, following initiatives undertaken by organisations of people with disabilities. The term ceased to be regarded as an individual problem or misfortune, but rather as a result of the interaction between features of person’s body and the society in which the person with a disability resides. [1] An inaccessible environment to a person with a disability can be the barrier to participation and inclusion [2]. According to the International Classification of Functioning, Disability and Health (ICF), there are three types of difficulties influencing human functioning: impairments, understood as problems in body function or alteration in body structure; activity limitations, creating difficulties in the execution of normal activities and, finally, participation restrictions related to problems with being involved in various aspects of life [3]. These three areas are interconnected and may affect the way a person with disability can access and participate in society. Those with disabilities frequently have limited capacities to participate in social life. Due to their impairments and limitations to their activity, they are additionally at risk of mental problems [4].

The development of the information society is perceived as an opportunity for a higher inclusion of persons with disabilities in social activities, but it can be another source of frustration and exclusion. Various studies have demonstrated that persons with disability experience a significant digital divide [5,6,7,8,9]. Therefore, the term, digital disability divide is used in reference to this population [6]. The population of people with disabilities is not homogenous, and the level of the digital divide varies according to the type of disability. It seems that the more severe the degree of disability, the lower is the use of the Internet [9,10]. However, the Pew Internet Study, performed in the USA in 2011, revealed that only 3% of those with disabilities indicated that “disability makes it very difficult, or impossible, for them to make use of the Internet” [7].

Therefore, the disability digital divide is not only an effect of the impairments occurring in people with disabilities, but frequently coexists with limitations to their social inclusion and their economic deprivation [6]. Living with disability leads to undesirable consequences, such as lower socioeconomic status, which in turn, restricts the use of information and communication technologies (ICT). Furthermore, many authors pointed out other limitations which preclude those with disabilities benefitting from making use of the Internet. Apart from the fact that disability itself, or related socioeconomic status, may result in an inability to use, or limit access to the Internet, the manner in which online services are designed may play a significant role. Frequently, the interfaces of online services are designed for young users and as a result, persons with various forms of disability can experience difficulties in using them [11,12]. It is also clear that significant efforts have been made to ensure access to the Internet for the general population. However, consideration of the level of computer and Internet use by people with disabilities has been frequently neglected, despite it being estimated that 16% the population experience some form of disability [2].

Those with disabilities may benefit from the use of the Internet in many ways. Their inclusion in social and professional activities is considered as one of the key benefits resulting arising from their ability to access the Internet and it may well enhance their general wellbeing. Although the modern approach to disabilities does not emphasise the medical background, the ability to tackle health issues is of the utmost importance, especially for the elderly population, in which disability is frequently a result of a long-term medical condition. It is also clear that the access to, and the use of, the Internet, ehealth and health literacy are closely interrelated [13,14].

The association of the use of the Internet by people having disabilities in Poland with wellbeing, mental health and health behaviours has not been studied in detail. Furthermore, such association have been rarely assessed in a nationally representative samples of these people. Although there are relatively extensive reports on the relationship between the Internet, wellbeing and mental health, much less is known about the relation of Internet use with the health behaviours of this disadvantaged group of the population. Taking advantage of the extensive dataset coming from the Social Diagnosis Study carried out biennially in Poland, the authors undertook an analysis of these relationships [15].

## 2. Materials and Methods

### 2.1. Overview

The analysis was based on the dataset available from the 2015 wave of the Social Diagnosis study performed in Poland biennially between 2000 and 2015 [15]. The methodology of the Social Diagnosis Study has been described earlier [16]. The dataset generated in 2015 is freely available to researchers who wish to undertake their own analyses. During the 2015 wave, 11,740 households and 26,308 individuals over 16 years old were interviewed [17]. From the generated dataset, the responses of those reporting a disability status were extracted. This may have been based on the decision of a relevant body, e.g., the Disability Evaluation Boards [18] or self-declared by persons who had not obtained any formal recognition of their status. The survey included items asking about the degree of disability but not its nature.

### 2.2. Statistical Analysis

The statistical analysis was carried out using the IBM SPSS v.21 (Armonk, NY, USA). All types of analysis on the data retrieved from the Social Diagnosis study were undertaken after making adjustments for the calibrated weights available from the study and included in the dataset. The survey methodology, sampling procedure and weighting system was described by the study team in relevant reports [16]. The variables included in the analysis were assessed with descriptive statistics. Frequencies were calculated for categorical variables and means and standard deviations for continuous variables. In the paper, the distribution of the responses to specific items was reported after exclusion of the missing values. The greatest level of missing values in the variables included in the analysis did not exceed 1.2%.

The assessment of the relation of the Internet with the variables reflecting the wellbeing, mental health and health behaviours of persons with disabilities was performed with multivariate logistic regression after adjustment for key sociodemographic variables and the degree of disability. The variables derived from the items related to the self-assessment of their own life and that enquired about the experience of loneliness were treated as indicators of wellbeing. Mental health was assessed on the basis of the responses to the items asking about suicidal thoughts and seeking psychological help. Finally, health behaviours included in the analysis included the undertaking physical activity, smoking tobacco and excessive alcohol consumption.

### 2.3. Logistic Regression Modelling

The regression modelling was preceded by a multicollinearity analysis. Both the variance inflation factor (VIF) and the tolerance remained within the recommended ranges (VIF values in the range from 1.030 to 1.605 and tolerance from 0.623 to 0.971). For all the models, the Hosmer and Lemeshow chi^2^ test and the Nagelkerke R square were calculated. For independent variables included in the regression models, odds ratio (OR) and 95% confidence intervals (95% CI) were provided.

The impact of Internet use was adjusted for key sociodemographic variables, including sex, age, place of residence (6 categories, from urban >500,000 inhabitants to rural); level of education (four levels, from primary to post-secondary non-tertiary or higher); professional activity (employee, entrepreneur or farmer; disability or retirement pension, pupil or student, vocationally inactive); marital status (married, single, widowed, separated) and for the degree of disability. The degree of disability was graded as mild, moderate, severe, or not established. Internet use was a dichotomous variable, with options only for a yes/no response.

Dependent variables included in the regression models were derived from items reflecting the wellbeing of the respondent, his or her mental health and health behaviours. The dependent variables were dichotomised, taking into consideration the distribution of the frequencies of the response categories and the explored features. In the results, the variables reflecting the self-assessment of their own life were dichotomized to compare the responses showing positive and non-positive feelings (1—for “very happy” or “rather happy”, and 0—for “not particularly happy” or “unhappy”). The variable related to suicidal thoughts was dichotomized to responses admitting (1—for “very often”, “quite often” or “rarely”) or not admitting to ever having such thoughts (0—for “never”). Other dependent variables were based on items requiring dichotomised responses in the questionnaire.

## 3. Results

### 3.1. Characteristics of the Study Group

The number of respondents included in the analysis after adjusting for the weights calculated by the Social Diagnosis study was 2529. The mean age (±standard deviation) of respondents was 59.33 ± 16.89 years; women 60.65 ± 17.41 and men 57.88 ± 16.19. Females comprised 52.24% (N = 1321), married persons 53.03% (N = 1337), persons on disability, or in receipt of a retirement pension 73.13% (N = 1847), residing in rural areas 40.13% (N = 917), and only 11.82% (N = 299) were educated to a post-secondary or university level. In the study group 20.71% (N = 524) of respondents had a mild disability, 41.58% (N = 1052) moderate and 26.54% (N = 671) suffered a severe disability. A total of 37.07% (N = 937) of the respondents were users of the Internet. A total of 67.19% (N = 1698) of the respondents assessed their life as happy or rather happy, 15.30% (N = 386) experienced suicidal thoughts at least rarely, 10.18% (N = 257) had received psychological help in the previous year, and 29.48% (N = 730) experienced unwanted loneliness. Some form of physical activity or sport was undertaken by only 21.61% (N = 536) of the respondents; 22.06% (N = 553) smoked cigarettes and excessive drinking of alcohol was admitted by 4.81% (N = 120). The characteristics of the study group is shown in Table 1.

### 3.2. Wellbeing

The self-assessment of the individual’s life was associated with their use of the Internet, age, marital status, professional activity, education level and the degree of disability (Table 2). Internet users tended to assess their life more favourably (OR, 95%CI: 1.86, 1.47–2.37). Greater happiness with life was also claimed by older persons with disabilities (0.990, 0.982–0.998) and respondents with a higher level of education (primary vs. upper secondary, 1.34, 1.03–1.74, primary vs. post-secondary non-tertiary or higher, 1.58, 1.10–2.26). Furthermore, married respondents were happier than single persons (0.44, 0.33–0.58), widowed (0.63, 0.39–0.81) and those having separated (0.34, 0.24–0.47). Vocationally active respondents claimed a happier life than those on retirement and in receipt of a disability pension (0.56, 0.40–0.80), or were unemployed (0.31, 0.21–0.45).

The use of the Internet was related to less frequently experiencing loneliness (0.63, 0.49–0.81). Married respondents were less prone to experience loneliness than those who were single (2.86, 2.16–3.78), widowed (2.98, 2.28–3.88), or separated (3.55, 2.54–4.97). Pupils and students experienced less loneliness than persons professionally active (0.40, 0.18–0.91). The feeling of loneliness was also dependent on the place of residence. Interestingly, persons living in urban areas with a population >500,000, were more prone to experience loneliness than those living in other urban areas or in rural areas. Furthermore, persons with a severe disability felt less lonely than those with a mild disability (0.67, 0.51–0.88).

### 3.3. Mental Health

The study found that persons with disability who had experienced suicidal thoughts were less frequently Internet users (0.47, 0.35–0.65) (Table 3). Suicidal thoughts were also more frequently admitted by respondents who were widowed (1.57, 1.12–20.19) or had separated (1.78, 1.20–2.65) than by those who were married. Suicidal thought were more often experienced by the unemployed than by professionally active persons (1.93, 1.21–3.08). The search for psychological help was associated with Internet use and professional activity. Seeking for psychological help was reported less frequently by those making use of the Internet (0.50, 0.35–0.72). However, it was searched more frequently by respondents who were retired or in receipt of a disability pension (2.03, 1.23–3.34) and those who were vocationally inactive (2.38, 1.39–4.08), but pupils and students searched for psychological help much less frequently (0.05, 0.002–0.83).

### 3.4. Health Behaviours

All three variables reflecting health behaviours (smoking excessive alcohol consumption and physical activity) depended on use of the Internet (Table 4). Persons with disabilities who used the Internet less frequently smoked (0.70, 0.54–0.91), less frequently declared excessive consumption of alcohol (0.32, 0.19–0.56) and more frequently participated in some type of physical activity or sport (2.41, 1.86–3.31). Older respondents less frequently smoked (0.97, 0.96–0.98), consumed alcohol excessively (0.98, 0.96–1.00) and participated in physical activity or sport (0.99, 0.976–0.995). Men, more than women, frequently smoked (2.19, 1.76–2.73) and excessively consumed alcohol (10.32, 5.54–19.26). Those living in urban areas with >500,000 residents more frequently smoked cigarettes than those living in urban areas of 20,000 or less inhabitants or in rural areas but also undertook physical activity more frequently than those living in rural areas (0.54, 0.37–0.78 and 0.64, 0.43–0.95, respectively).

Physical activity was also more frequently declared by persons with higher levels of education than those with only primary education. Finally, health behaviours were also related to professional activity. Respondents who were professionally active less frequently smoked than those who were professionally inactive (1.81, 1.25–2.61), and more frequently undertook some form of physical activity (0.54, 0.36–0.81). However, the professionally active respondents more frequently drank alcohol excessively than those in retirement, or on in receipt of a disability pension (0.49, 0.27–0.90).

## 4. Discussion

The data obtained from 2529 persons with disabilities included in the 2015 wave of the Social Diagnosis study were analysed. In the group, only 37.1% were Internet users (32.9% after applying weights from the study), compared to 66.1% of the general population [17]. This is in agreement with many previous studies which have reported much lower use of the Internet by persons with disabilities than by the general population [7,9,19,20,21].

The analysis revealed that the use of the Internet, after adjusting for sociodemographic variables and the degree of disability, demonstrated a significant relationship with variables reflecting the wellbeing, mental health and health behaviours of persons with disabilities. The odds that Internet users confirm that their life was very or quite happy was two times higher than for non-users. Internet users had also a 40% lower risk of experiencing loneliness and 50% lower chance of experiencing suicidal thoughts or seeking for psychological help than non-users. In addition, the likelihood of Internet users smoking tobacco was 30% lower than non-users and the excessive consumption of excessive alcohol was as much as 70% lower than for those who did not use the Internet. Furthermore, it was shown that people with disabilities who used the Internet were nearly 2.5 times more likely to perform some form of physical activity or participate in sport than non-users. These findings tend to confirm the overall association of the Internet use with various positive aspects of life of persons with disabilities.

Early studies assessing the relation of the Internet with the wellbeing and mental health of the general population yielded unequivocal results. Some authors indicated that Internet usage resulted in a shorter time spent in face-to-face contact with others and was therefore harmful to interpersonal relationships [22,23]. Kraut et al. also reported a positive correlation between Internet use and depression, loneliness and stress [22]. They even reported on an “Internet paradox” which indicated that “social technology” reduces the social involvement and psychological wellbeing of Internet users. Sanders et al. observed similar effects among adolescents [23].

However, other reports showed that beneficial effects resulted from having access to and use of the Internet. Surveys carried out in the USA at the end of 20th century showed that Internet users appreciated the ability to maintain contact with friends and families [24,25]. In 2002, Shaw and Gant reported that Internet use resulted in significantly lower levels of loneliness and depression and increased the perceived degree social support and self-esteem [26]. Discussions about the net effects of Internet use have evolved in last 20 years, in line with rapid growth of the number of Internet users and the new tools that facilitate interaction and social networking. It is also clear that definite responses cannot be expected. The impact of the Internet depends on the population that uses it, the intensity of its use and the types of online resources included in the analysis.

From the early stages of the growth of Internet use, there were authors who claimed that the Internet would allow disadvantaged people to overcome their limitations [27,28,29]. According to Bradley and Poppen, persons with disabilities who used the Internet considered that the level and quality of their communication was enhanced [30]. Other authors reported an improved sense of independence and self-determination in persons with disabilities who used the Internet [31,32]. Guo et al. confirmed the existence of the digital divide for the disabled community in China, but simultaneously indicated that although it was a minority of people with disabilities who had access to the Internet, they gained considerable benefits, based on the frequency and quality of their social interaction [20]. The impact of the use of the Internet by people with disabilities has been frequently analysed on the basis of the compensation model proposed by Birnie & Horvath in 2002 [33]. According to this model, persons socially inactive in the real world may more frequently use and benefit from the Internet. For persons with disabilities, online modes of communication may be the way to compensate their low level of social interaction.

In 2011, Cheatham published a review of the studies focusing on the effects of Internet use on the wellbeing of adults with physical disabilities. Apart from admitting that there were not many outcome studies in this areas, he reported that in three of the six studies considered, the relationship between use of the Internet and wellbeing was statistically significant [34]. Smedema and McKenzie (2010) reported a positive association between Internet use and the overall sense of well-being in a sample of 175 persons with visual impairments [35]. Using online chat was positively associated with social support and well-being. However, disability-related information seeking and participation in online support groups exerted a negative effect on wellbeing in this group. Shpigelman and Gill carried out a survey among 172 respondents on how persons with various types of disabilities used Facebook [36]. The most important benefits indicated by the respondents included the possibility of communicating with friends, the ability to choose with whom to talk and the possibility of spending and enjoying leisure time online.

There is also evidence that persons with intellectual disabilities may gain benefits from the use of ICT. In a review published in 2016, Caton analysed the impact of the use of social media by people with intellectual disabilities [37]. The benefits identified included positive social and emotional experience in the context of friendship, the development of a social identity and self-esteem [37]. Other authors also indicated an increased level of social interactions, connectedness, participation in mutual support groups and access to information [38,39].

Recently, Lee et al. focused on the effects arising from the use of social network sites (SNS) and online communities on those with physical disabilities [40]. In their paper published in 2019, they reported that a higher intensity of use of SNS and greater contact with the online community led to lower levels of depression. Their study was based on the survey of 91 users of SNS and online communities. After analysing the interviews collected from a group of 15 subjects, they found that the use of social media helped them to build social support and health psychological dispositions. The results of the systematic literature review performed by Sweet et al. revealed that there are many reports indicating the positive aspects of social media use by people with disabilities, including gaining knowledge, forming friendships and creating social support groups [41].

There is also a mounting evidence that the use of ICT may also have a preventive influence on the development or progression of dementia in the elderly population [42,43,44]. As the elderly constitute the major part of the population suffering disabilities, such findings may be a further argument for supporting the use of computer and the Internet by the elderly. Interesting results have been reported recently by Jin et al. [45]. They analysed the data from a longitudinal study encompassing a cohort of 13,457 Chinese adults. They concluded that desktop and cell phone ownership, after adjusting for demographic variables, health behaviour and health risk factors, were independently associated with lower cognitive decline.

There are many studies assessing the effects of Internet use on the wellbeing and mental health of the elderly. As, in most societies, it is older people who comprise the overwhelming portion of those with disabilities, these studies can be used to infer the effect of using the Internet by all persons with disabilities. Cotten et al. used regression methods to analyse the data of 205 participants from ongoing randomized controlled trial on ICT intervention. They found that Internet use was positively associated with mental wellbeing and reduced symptoms of depression [46]. In the prospective study carried out by Hamer and Stamatakis on 6359 elderly persons from the English Longitudinal Study of Ageing, Internet users reported lower depressive symptoms and higher levels of global cognitive function [47].

However, there are also studies which do not indicate benefits and some which even suggest that Internet use is harmful. For example, Miller found that in a group of 137 people with a spinal cord injury, Internet use was not related significantly to any perceived social support [48]. Furthermore, Internet use showed a significant negative effect on the overall sense of wellbeing. The frequency of online gaming was negatively associated with measures used for assessing wellbeing. In turn, seeking disability-related information was negatively associated with psychological and financial wellbeing as well as perceived social support. Additionally, a study carried out by Jenaro et al. in the group of 216 young adults with intellectual disabilities revealed that they tend to exploit the more social and recreational features of the Internet and mobile phones than the educational functionalities and more frequently make excessive use of them when compared to their peer group without disabilities [49]. The overuse of these tools was also associated with other unhealthy behaviours.

In this study, we did not analyse specific types of disabilities, but explored the responses of all persons with disability included in national Social Diagnosis study. Nevertheless, the findings confirm the strong association of using the Internet with the wellbeing and mental health of those with disabilities. The positive relationship was seen also in case of health behaviours. The respondents who used the Internet were more likely to undertake some form of physical activity and were less at risk of smoking or excessive drinking of alcohol. It is apparent that, to date, the association of Internet use with the health behaviours in the population of persons with disabilities has not been widely considered.

Some authors observed that persons with disabilities may gain health-related benefits from the use of the Internet—not precisely in relation to health behaviours, but rather to health-related quality of life or coping with medical conditions. In a review published in 2004, Magnusson et al. reported that the use of ICT by elderly individuals with disabilities was associated with a reduced length and frequency of hospitalisation [50]. In turn, Drainoni et al. observed that persons with spinal cord injuries who used the Internet had an improved health-related quality of life [51]. A survey was undertaken on 516 participants of 16 national Model Spinal Cord Injury Systems programmes in the USA.

In our study, logistic regression modelling revealed that Internet use, after adjusting for the set of sociodemographic variables and the degree of disability, was associated with dependent variables reflecting wellbeing, mental health and health behaviours. Some of these variables were also associated with dependent variables. Respondents who were professionally active more often declared happiness with life, less frequently experienced loneliness and suicidal thoughts, and less frequently searched for psychological help than those in other categories of vocational status. Furthermore, professionally active respondents less frequently smoked and more frequently undertook some form of physical activity. These finding appear to be in line with the modern approach to chronic illness and disability. Providing job opportunities and removing occupational barriers have been identified as one of the key strategies for the promotion of wellbeing and social integration of people with disabilities. The positive effect of employment on satisfaction with life has been reported, even in persons with the most severe types of disabilities. The survey performed by Dijkers on 2183 persons with spinal cord injury revealed that being vocationally active was a predictor of higher life satisfaction [52]. Subjective wellbeing (SBW) was assessed by Uppal based on a sample of 24,036 Canadians with disabilities [53]. He demonstrated that SWB measured by the level of happiness did not depend on per capita family income, but it was lower among the unemployed. The results of the systematic review published in 2018 by Lindsay et al. confirmed that the benefits from remaining employed for people with disabilities, apart from improved income, included a higher quality of life, enhanced self-confidence, an expanded social network and a sense of community [54].

In our study, married respondents were happier and less frequently experienced loneliness and suicidal thoughts. The analysis reported by Bardo in 2015 showed that marital status was related to increased happy life expectancy among people with physical disabilities [55].

We also found that older persons with disabilities felt happier than younger people with disabilities, less frequently smoked and drank alcohol excessively. Gove et al. reported that in general, an older population was related to higher life satisfaction [56]. However, other studies showed that happiness rose in midlife and declined slowly thereafter [57]. In Uppal’s analysis of data obtained from the survey of about 24,000 Canadians with disabilities, the relationship between happiness and age was U-shaped, with the lowest values occurring at around 40 years of age [53]. A higher life satisfaction by older people with disabilities than by younger disabled people was also reported by Addabbo et al. on the basis of an analysis of the data of 3121 respondents participating in a national survey undertaken in Italy [58]. Lower rates of smokers among older persons with disabilities were also reported earlier [59].

Overall, however, people with disabilities more frequently smoked cigarettes, were overweight or obese and less frequently undertook physical activity than people without disabilities [60,61,62,63]. Our study revealed that men demonstrated much more harmful health behaviours than women. They smoked cigarettes twice as often and 10 times more frequently confirmed excessive alcohol consumption than women. Similar trends are also seen in the general population [64,65].

It is also worthy of note that persons living in urban areas with populations of 500,000 and over more frequently experienced loneliness, more frequently smoked, but also more frequently undertook physical activity than those with disabilities living in smaller cities or rural areas. Respondents who had attained higher levels of education than a primary were happier and more frequently declared some form of physical activity.

In our study, variables modelled in logistic regression did not depend on the degree of disability. Such findings were not expected, as earlier reports tended to show lower levels of happiness by people having more severe disabilities [53,58].

### Limitations

The analysis of the data originating from the survey did not enable an understanding of the direction of the causal effect to be obtained. However, the fact that use of the Internet was consistently related to indicators of improved wellbeing, mental health and even to more beneficial health behaviours may be seen as the positive influence of the Internet for persons with disabilities. In the study, the mechanisms responsible for the reported effects were not analysed. Judging from the results of other studies, social networking, enhanced communications with family, friends, or peers; greater access to various types of information, education and entertainment may be probable mediators improving the wellbeing and providing support for the mental health of this population. It also seems obvious that persons with milder forms of disabilities have a greater chance to use the Internet and other forms of ICT. In the regression models that were applied in the study, the positive effects related to the Internet use persisted after adjusting for the degree of disability and other factors, including the key sociodemographic variables.

It has not been possible to provide more insights into the relationship between Internet use and the dependent variables across the types of disabilities. The survey was not focused specifically on disability and the items focusing on the nature of disabilities were not included in the questionnaire. This aspect could certainly be addressed in future studies.

## 5. Conclusions

Even now, nearly 30 years after the start of the Internet expansion, people with disabilities still experience a significant digital divide. However, those who are able to access the Internet may benefit considerably. Their wellbeing, mental health and even health behaviours seem to be more favourable than those who do not use the Internet. This effect is maintained after adjustment for sociodemographic variables and the degree of the disability. These results may be another argument for intensifying actions to enable persons with disabilities to obtain access to the Internet and, if possible, accompany the actions with supportive educational interventions aimed at improving their digital literacy.

## Figures and Tables

**Table 1:** Characteristics of the study group.

Variable	Response Option	Unweighted		Weighted	
		*N*	%	*N*	%
Sex	female	1543	54.56	1321	52.24
	male	1285	45.44	1208	47.76
Marital status	married	1591	56.48	1337	53.03
	single	432	15.34	492	19.51
	widowed	582	20.66	509	20.19
	in separation	212	7.53	183	7.27
Professional activity	employee or entrepreneur or farmer	309	10.95	333	13.17
	retired or disability pension	2178	77.15	1847	73.13
	pupils or student	41	1.45	45	1.80
	unemployed or vocationally passive from other reasons	295	10.45	301	11.90
Place of residence	urban >500,000	167	5.91	243	10.63
	urban 200,000–500,000	241	8.52	278	12.15
	urban 100,000–200,000	208	7.36	208	9.11
	urban 20,000–100,000	604	21.36	561	24.54
	urban below 20,000	360	12.73	322	14.07
	rural	1248	44.13	917	40.13
Education	primary	841	29.78	696	27.56
	lower secondary	942	33.36	861	34.09
	upper secondary	735	26.03	670	26.53
	post-secondary non-tertiary or higher	306	10.84	299	11.82
Degree of disability	mild	620	21.92	524	20.71
	moderate	1127	39.85	1052	41.58
	severe	752	26.59	671	26.54
	not established	329	11.63	282	11.17
Internet use	no	1897	67.15	1590	62.93
	yes	928	32.85	937	37.07
Taking into consideration all together, how would you assess your life?	not particularly happy or unhappy	968	34.27	829	32.81
	very or quite happy	1857	65.73	1698	67.19
How often in last months, it happened you felt so broken that you thought about suicide	never	2385	84.54	2137	84.70
	very or quite often or rarely	436	15.46	386	15.30
In last year, I used the advice from psychologist (psychiatrist)	no	2561	90.65	2270	89.82
	yes	264	9.35	257	10.18
Do you experience loneliness despite you do not want to be alone?	no	1990	72.08	1747	70.52
	yes	771	27.92	730	29.48
In last year, I have drunk to much alcohol	no	2696	95.57	2385	95.19
	yes	125	4.43	120	4.81
Do you smoke?	no	2226	78.77	1955	77.94
	yes	600	21.23	553	22.06
Do you undertake some form of physical activity or sport	no	2242	80.07	1944	78.39
	yes	558	19.93	536	21.61

**Table 2:** Logistic regression modelling for variables reflecting the wellbeing of respondents.

Independent Variables	Self-Assessment of Own Life ^1^			Experiencing Loneliness ^2^		
	OR	95%CI	*p*	OR	95%CI	*p*
Sex	1.08	0.89–1.31	0.435	0.93	0.76–1.14	0.473
Marital status						
married						
single	0.44	0.33–0.58	<0.001	2.86	2.16–3.78	<0.001
widowed	0.63	0.49–0.81	<0.001	2.98	2.28–3.88	<0.001
in separation	0.34	0.24–0.47	<0.001	3.55	2.54–4.97	<0.001
Professional activity						
employee, entrepreneur or farmer						
retired or disability pension	0.56	0.40–0.80	0.001	1.06	0.77–1.47	0.71
pupil or student	1.52	0.59–3.88	0.38	0.40	0.18–0.91	0.029
unemployed or vocationally passive from other reasons	0.31	0.21–0.45	<0.001	1.44	0.99–2.09	0.059
Age	0.99	0.982–0.998	0.023	0.997	0.98–1.01	0.48
Place of residence						
urban >500,000						
urban >200,000–500,000	0.96	0.65–1.41	0.82	0.51	0.35–0.76	0.001
urban >100,000–200,000	1.15	0.75–1.76	0.52	0.64	0.42–0.98	0.040
urban >20,000–100,000	1.05	0.75–1.48	0.76	0.59	0.42–0.83	0.003
urban <=20,000	1.18	0.81–1.73	0.39	0.54	0.37–0.79	0.002
rural	1.30	0.93–1.81	0.12	0.51	0.36–0.71	<0.001
Education level						
primary						
lower secondary	0.88	0.70–1.12	0.30	1.18	0.92–1.50	0.18
upper secondary	1.34	1.03–1.74	0.027	0.78	0.59–1.02	0.068
post-secondary non-tertiary or higher	1.58	1.10–2.26	0.013	0.91	0.63–1.30	0.60
Degree of disability						
mild						
moderate	1.01	0.79–1.28	0.94	0.85	0.66–1.08	0.19
severe	1.23	0.94–1.60	0.13	0.67	0.51–0.88	0.004
not established	0.76	0.55–1.05	0.098	1.04	0.74–1.45	0.82
Internet use	1.86	1.47–2.37	<0.001	0.63	0.49–0.81	<0.001
Constant	5.76		<0.001	0.66		0.26

^1^ Hosmer-Lemeshow test chi^2^ = 14.428, *p* = 0.07, Nagelkerke R^2^ = 0.13; ^2^ Hosmer-Lemeshow test chi^2^ = 23.824, *p* = 0.002, Nagelkerke R^2^ = 0.13; OR—odds ratio, 95%CI—95% confidence interval.

**Table 3:** Logistic regression modelling for variables reflecting the mental health of respondents.

Independent Variables	Suicidal Thoughts ^1^			Seeking for Psychological Help ^2^		
	OR	95%CI	*p*	OR	95%CI	*p*
Sex	1.24	0.97–1.58	0.090	0.88	0.65–1.17	0.37
Marital status						
married						
single	1.00	0.70–1.43	0.99	1.17	0.79–1.74	0.42
widowed	1.57	1.12–2.19	0.009	0.62	0.37–1.03	0.063
in separation	1.78	1.20–2.65	0.004	0.87	0.50–1.54	0.64
Professional activity						
employee, entrepreneur or farmer						
retired or disability pension	1.42	0.93–2.16	0.10	2.03	1.23–3.34	0.006
pupil or student	0.74	0.27–2.03	0.56	0.05	0.002–0.83	0.037
unemployed or vocationally passive from other reasons	1.93	1.21–3.08	0.006	2.38	1.39–4.08	0.002
Age	0.98	0.97–0.99	<0.001	0.96	0.94–0.97	<0.001
Place of residence						
urban >500,000						
urban >200,000–500,000	0.57	0.34–0.97	0.039	1.01	0.53–1.93	0.98
urban >100,000–200,000	1.03	0.61–1.72	0.92	1.08	0.54–2.14	0.82
urban >20,000–100,000	0.95	0.62–1.45	0.81	1.32	0.76–2.28	0.33
urban <=20,000	0.91	0.57–1.46	0.71	0.66	0.34–1.28	0.22
rural	0.73	0.49–1.11	0.14	0.93	0.54–1.59	0.78
Education level						
primary			0.75			0.99
lower secondary	1.01	0.75–1.35	0.95	1.01	0.71–1.45	0.94
upper secondary	0.91	0.65–1.26	0.57	1.04	0.69–1.57	0.85
post-secondary non-tertiary or higher	1.14	0.73–1.77	0.57	1.09	0.63–1.90	0.76
Degree of disability						
mild						
moderate	1.15	0.84–1.57	0.37	1.06	0.73–1.55	0.75
severe	1.24	0.88–1.74	0.22	1.38	0.92–2.08	0.12
not established	1.12	0.73–1.74	0.60	0.95	0.54–1.69	0.87
Internet use	0.47	0.35–0.65	<0.001	0.50	0.35–0.72	<0.001
Constant	0.56		0.21	0.96		0.95

^1^ Hosmer-Lemeshow test chi^2^ = 8.883, *p* = 0.35, Nagelkerke R^2^ = 0.06; ^2^ Hosmer-Lemeshow test chi^2^ = 9.936, *p* = 0.27, Nagelkerke R^2^ = 0.13; OR—odds ratio, 95%CI—95% confidence interval.

**Table 4:** Logistic regression modelling of variables associated with health behaviours.

Independent Variables	Smoking ^1^			Excessive Alcohol Consumption ^2^			Physical Activity ^3^		
	OR	95%CI	*p*	OR	95%CI	*p*	OR	95%CI	*p*
Sex	2.19	1.76–2.73	<0.001	10.32	5.54–19.26	<0.001	1.22	0.98–1.53	0.074
Marital status									
married									
single	0.70	0.51–0.96	0.029	0.86	0.49–1.53	0.62	1.03	0.75–1.43	0.85
widowed	1.12	0.81–1.56	0.49	0.30	0.08–1.16	0.080	0.87	0.61–1.24	0.43
in separation	2.18	1.54–3.09	<0.001	1.32	0.69–2.51	0.40	1.21	0.83–1.78	0.33
Professional activity									
employee, entrepreneur or farmer									
retired or disability pension	0.92	0.66–1.26	0.59	0.49	0.27–0.90	0.021	0.89	0.65–1.21	0.45
pupil or student	0.08	0.02–0.41	0.002	1.50	0.37–6.05	0.57	1.55	0.76–3.15	0.23
unemployed or vocationally passive from other reasons	1.81	1.25–2.61	0.002	1.73	0.93–3.22	0.085	0.54	0.36–0.81	0.003
Age	0.97	0.96–0.98	<0.001	0.98	0.96–1.00	0.049	0.99	0.976–0.995	0.003
Place of residence									
urban >500,000									
urban >200,000–500,000	0.87	0.57–1.33	0.53	1.48	0.59–3.69	0.40	0.93	0.60–1.43	0.73
urban >100,000–200,000	0.67	0.42–1.08	0.10	0.43	0.11–1.67	0.22	0.68	0.42–1.11	0.12
urban >20,000–100,000	0.76	0.52–1.10	0.15	1.51	0.66–3.43	0.33	1.43	0.98–2.08	0.066
urban <=20,000	0.60	0.39–0.92	0.020	0.99	0.39–2.49	0.98	1.22	0.80–1.87	0.35
rural	0.54	0.37–0.78	0.001	0.77	0.33–1.77	0.53	0.64	0.43–0.95	0.026
Education level									
primary									
lower secondary	1.05	0.81–1.37	0.71	0.60	0.36–0.97	0.039	1.38	1.00–1.90	0.047
upper secondary	0.78	0.58–1.06	0.11	0.79	0.43–1.44	0.44	1.58	1.13–2.21	0.007
post-secondary non-tertiary or higher	0.80	0.54–1.20	0.28	1.05	0.48–2.29	0.91	2.40	1.63–3.55	<0.001
Degree of disability									
mild									
moderate	0.78	0.61–1.01	0.059	0.77	0.48–1.22	0.26	0.93	0.71–1.21	0.58
severe	0.59	0.44–0.80	0.001	0.27	0.14–0.53	<0.001	0.55	0.40–0.76	<0.001
not established	0.51	0.34–0.77	0.002	0.57	0.27–1.22	0.15	0.96	0.65–1.41	0.83
Internet use	0.70	0.54–0.91	0.007	0.32	0.19–0.56	<0.001	2.41	1.86–3.13	<0.001
Constant	2.09		0.061	0.12		0.012	0.39		0.019

^1^ Hosmer-Lemeshow test chi^2^ = 6.675, *p* = 0.57, Nagelkerke R^2^ = 0.13; ^2^ Hosmer-Lemeshow test chi^2^ = 5.192, *p* = 0.74, Nagelkerke R^2^ = 0.24; ^3^ Hosmer-Lemeshow test chi^2^ = 12.039, *p* = 0.15, Nagelkerke R^2^ = 0.19; OR—odds ratio, 95%CI—95% confidence interval.
